# Daptomycin: Local Application in Implant-Associated Infection and Complicated Osteomyelitis

**DOI:** 10.1100/2012/578251

**Published:** 2012-06-18

**Authors:** Steffen B. Rosslenbroich, Michael J. Raschke, Carolin Kreis, Nancy Tholema-Hans, Andreas Uekoetter, Rudolf Reichelt, Thomas F. Fuchs

**Affiliations:** ^1^Department of Trauma, Hand, and Reconstructive Surgery, University Hospital Muenster, Westfalian-Wilhelm's-University Muenster, Waldeyerstraße 1, 48149 Muenster, Germany; ^2^Institute of Medical Microbiology, University Hospital Muenster, Westfalian-Wilhelm's-University Muenster, Domagkstraße 10, 48149 Muenster, Germany; ^3^Institute of Medical Physics and Biophysics, University Hospital Muenster, Westfalian-Wilhelm's-University Muenster, Robert-Koch-Straße 31, 48149 Muenster, Germany

## Abstract

*Background*. The rise of highly resistant bacteria creates a persistent urge to develop new antimicrobial agents. This paper investigates the application of the lipopeptide antibiotic daptomycin in infections involving the human bone. *Methods*. Compressive and tensile strength testing of daptomycin-laden PMMA was performed referring to the ISO 5833. The microstructure of the antibiotic-laden PMMA was evaluated by scanning electron microscopy. Intracellular activity of daptomycin was determined by a human osteoblast infection model. Elution kinetics of the antibiotic-laden bone cement was measured by using a continuous flow chamber setup. *Results*. There was no significant negative effect of adding 1.225% and 7.5% per weight of daptomycin to the PMMA. There was no significant difference in intracellular activity comparing gentamicin to daptomycin. Elution of daptomycin from PMMA showed within the first-hour initial peak values of 15–20 **μ**g/mL. *Conclusion*. Daptomycin has a certain degree of activity in the intracellular environment of osteoblasts. Daptomycin admixed to PMMA remains bactericidal and does not significantly impair structural characteristics of the PMMA. The results of this paper suggest that daptomycin might be a potent alternative for treating osteomyelitis and implant-associated infection in trauma and orthopedic surgery caused by multiresistant strains.

## 1. Introduction

Although the rate of infection following joint replacement surgery is relatively low [1–3], 0.5–3% are reported, revisions of prostheses and internal fixation of open fractures are associated with an estimated rate of infection of up to 40% [3, 4]. The presence of traumatized and scarred tissue, bone necrosis, and foreign body surfaces, as well as further general predisposing factors, for example, diabetes and peripheral vascular disease, which impair the host defense system, tip the balance in favor of infection, [3–8]. The gram-positive bacteria, *Staphylococcus aureus *and* S. epidermidis*, are the major causative agents of the bone disease osteomyelitis [1, 3, 5, 6, 9–11]. Attachment of these bacteria to foreign body surfaces such as endoprostheses or internal devices induces the synthesis of a bacterial extracellular polysaccharide matrix [3]. The biofilm acts as a barrier for antibacterial compounds and the hosts immune system, while simultaneously bacteria in a biofilm have a reduced metabolic rate, thus rendering the eradication of the infective pathogens difficult, especially by growth-dependent antibiotics [3, 4, 12, 13]. Furthermore, the capability of *S. aureus *to avoid the host immune system into the osteoblast's intracellular environment [14–17], thus creating a reservoir of bacteria for recurring osteomyelitis [5, 6], might be more relevant to chronic disease than bacteria associated with the bone matrix. For systemically applied antibiotics, penetration and diffusion barriers exist such as biofilms, tissue changed by inflammation or necrotic areas. Pathological changes often require high concentrations of the active substance at the site of infection, but even high and potentially toxic doses of active substances cannot always ensure adequate levels of activity at the area of need [18]. If, however, the antibiotic is implanted directly into the site of infection by means of an adequate carrier, high concentrations of the active substance could be achieved without systemic toxic side effects. Local application of antibiotics has become a standard procedure in treatment of osteomyelitis and implant-associated infection in musculoskeletal surgery.

For local release of antibiotics, currently antibiotic-impregnated collagen sponges, antibiotic-coated devices, antibiotic-loaded calcium sulfate pellets, and polymethylmethacrylate beads are implanted [19–22]. Antibiotic-loaded spacers provide contemporaneous stabilization and spacing but primarily infection treatment to the region of interest through high local concentration and continuous release of the antibiotic substance without the need for multiple revisions.

Gentamicin and tobramycin are two aminoglycosides commonly impregnated into polymethylmethacrylate for treatment and prevention of implant-associated and prosthetic joint infection [21, 23, 24]. However, the isolation of gentamicin-resistant bacterial isolates from infected prosthetic hip joints has raised concerns about the routine use of gentamicin-loaded bone cement [25]. Furthermore, growth of *Staphylococcus* spp. isolates was reported on gentamicin-loaded cement beads, and a nonclinical *S. aureus* isolate has also shown to form biofilms on a number of different gentamicin-loaded bone cements despite presence of the antibiotic [25–27].

Additionally infections due to methicillin-resistant *Staphylococcus aureus* (MRSA) are increasingly common in nosocomial and community settings [21, 28, 29], being more likely to be resistant to gentamicin or tobramycin than their methicillin susceptible counterparts, thus further limiting treatment options posing a major problem throughout the world [6, 30]. The glycopeptide vancomycin was once the antibiotic of choice to treat resistant strains, but certain strains also developed resistance to this agent [20, 21, 30–33] reducing its application in multiresistant infections. These findings implicate the need for further agents other than amino glycosides and glycopeptides for the treatment and prophylaxis of prosthetic joint and implant-associated infection [20, 23].

One of these agents might be the cyclic lipopeptide daptomycin, an antibiotic with rapid bactericidal activity against *S. aureus *[29, 34–36]. It has shown a high *in vivo* activity against MRSA and is currently approved for treatment of complicated skin and soft tissue infections, bacteremia, and right-sided endocarditis [21, 37–39]. The mode of antimicrobial action is not yet entirely clear. However, daptomycin is supposed to inhibit peptidoglycan biosynthesis, either directly or indirectly, and to promote membrane depolarization [40].

This agent should be useful in systemic or local treatment of osteomyelitis [21, 41, 42] caused by gram-positive pathogens since there are few reports of resistance so far. Daptomycin has been shown to penetrate into bone [43] as well as it is reported to be effective against both actively growing and stationary-phase pathogens embedded in biofilms [20, 39, 44–46]. There is some evidence for intracellular accumulation of daptomycin [47, 48] but concerning the intracellular activity, especially in osteoblasts, current literature provides only little information.

To determine whether daptomycin is applicable for the clinical use in prophylaxis and treatment of osteomyelitis and implant-associated infections, its intracellular activity and the feasibility of mixing daptomycin in polymethylmetacrylate (PMMA) have to be taken into consideration. There are several aspects when mixing an antibiotic with PMMA, which could cause alteration especially in respect to structural properties of the PMMA and in the biological efficacy of the applied antibiotic. These possible alterations might be due to exothermic reactions after mixture of the two components of PMMA-cement where temperatures up to 60°C are reported possibly changing antibiotic efficacy. Furthermore, changes in the mixing ratio caused by adding the antibiotic to one of the components of the PMMA or unintentional intermaterial reactions might have influence on its properties.

Aim of this study was to evaluate the extra and intracellular antimicrobial efficiency of daptomycin.

In order to answer this question, elution kinetics and structural properties of daptomycin loaded bone cement were evaluated. In addition, the agent was investigated for its options for treating infections in orthopedic and trauma surgery. 

## 2. Material and Methods

### 2.1. *In Vitro* Infection

Due to the fact that* S. aureus *has the capability to move into the osteoblast's intracellular environment [14–17], a primary human osteoblast infection model modified by Van der Auwera et al. [48] was used to assess intracellular activity of daptomycin.

Primary human osteoblasts were routinely cultured in growth medium consisting of Minimum Essential Medium (Eagle) (MEM) and HAM's F-12 (1 : 1), supplemented with 10% fetal calf serum (FCS), 100 U of penicillin mL^−1^, and 100 *μ*g of streptomycin mL^−1^. All samples were obtained with permission of the local ethic committee. The assay medium was MEM/HAM's F-12 (1 : 1), supplemented with 1% human serum albumine (HSA). Prior to assay [49], bacteria (different* Staphylococcus *strains with different degrees of intracellular invasiveness; *Cowan, TM 300, 49230*) were grown overnight. Bacterial cell numbers were estimated spectrophotometrically at 540 nm. Bacteria were harvested by centrifugation (5 min, 4°C, 5000 rpm) and resuspended in 1 mL phosphate-buffered saline pH 7.3 (PBS) supplemented with 1% HSA to give 5 × 10^8^ CFU mL^−1^. Cells were seeded at 10^5^ per well into 12-well tissue cultures plates in 1 mL of growth medium. Accordingly, they were cultured for 2 days until they were confluent; at the first day of assay, cells were washed twice with 1 mL of MEM/HAM's F-12 and then incubated with 1 mL of assay medium. 40 *μ*L of the bacteria culture with an OD_540 nm_ =1 (= 5 × 10^8^ CFU mL^−1^) were added to the osteoblasts, incubated for 30 minutes at 25°C to allow sedimentation and then shifted to 37°C in a 5% CO_2_ incubator for 3 h. After the 3 hours, coculture of bacteria (multiplicity of infection [MOI] of 100 : 1) external *S*. *aureus* was inactivated by 20 *μ*g mL^−1^ lysostaphin. To determine success of internalization, cells were washed subsequently with 1 mL of PBS to remove noninternalized bacteria, stripped of the wells by adding 0.2 mL of 0.05% Trypsin containing 0.02% EDTA and stopped with 0.8 mL MEM/HAM's F-12 and 10% FCS. After washing twice with PBS, bacteria were harvested via lysis by adding 1 mL aqua dest, and cell enumeration of colony-forming units (CFUs) was performed by serial dilution and plate counting on Mueller-Hinton agar plates. Alternatively after the lysostaphin treatment, osteoblasts cultures were washed twice with 1 mL of MEM/HAM's F-12 to remove noninternalized bacteria, 1 mL of fresh growth medium containing gentamicin (10 *μ*g mL^−1^), daptomycin (10 *μ*g mL^−1^), or rifampicin (7 *μ*g mL^−1^) or the combination daptomycin/gentamicin or daptomycin/rifampicin instead of penicillin/streptomycin were added, and the cultures were incubated for up to 20 and 40 hours. Internalization of *S. aureus* was quantified as described above. The invasiveness of Cowan I was set at 100%, TM 300 with no invasiveness served as the negative control group [49].

### 2.2. Biomechanics

To decide whether the biomechanical characteristics of certain types of bone cement are sufficient for clinical use, standardized tests are necessary. These tests are summarized in the ISO 5833 edited by the International Organization for Standardization. The ISO 5833 specifies the physical, mechanical, packaging, and labeling requirements for curing polymerizing radio-opaque and non-radio-opaque resin cements based on polymethacrylic acid esters [50]. Concerning biomechanical requirements, the ISO 5833 interalia stipulates standardized test-settings to determine the cement's compressive strength, bending modulus, and bending strength.

### 2.3. Compressive Strength

Cylinders of 12 (±0.1) mm in length and 6 (±0.1) mm in diameter are produced by using specially fabricated forms made of stainless steel and mixing the components under vacuum as described in the manufactures instructions (Palacos R, Heraeus).

After filling the two-component PMMA in the cavities of the form, pressure is applied for 60 minutes in a bench vice allowing the cement to set. Special devices are used to loosen the cylinders subsequently measuring and grinding to exact size if necessary. Thereafter, the samples are stored for 24 hours at 23 (±1)°C to completely harden.

**Figure 1:**
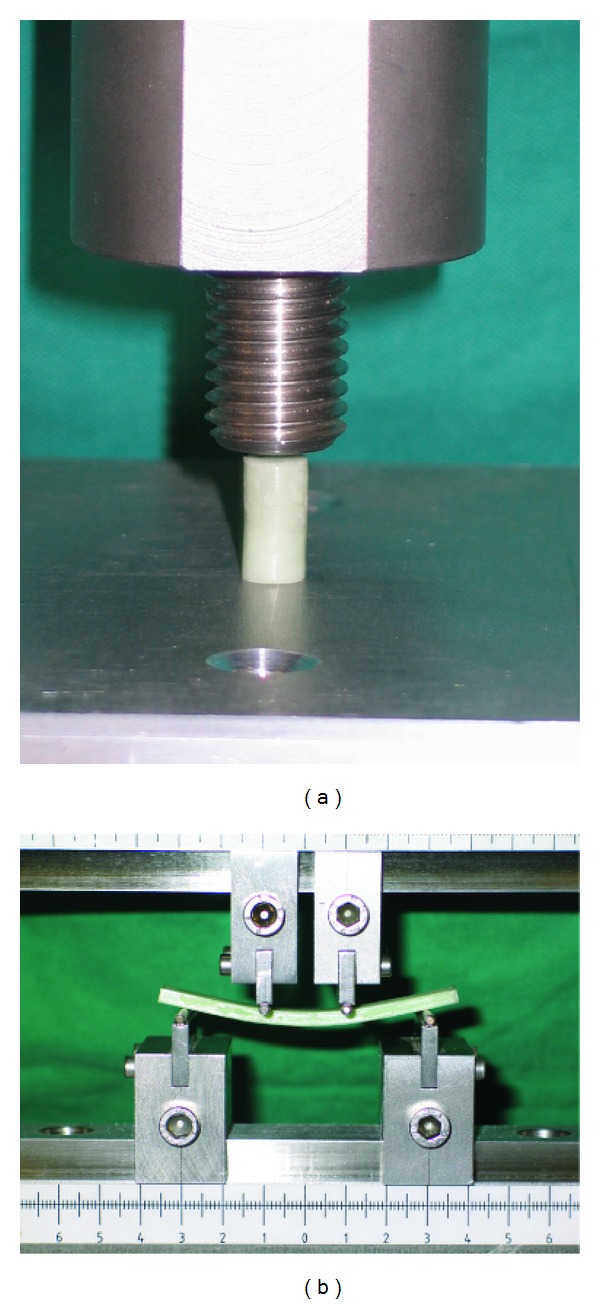
Test setup for (a) compressive strength testing and (b) bending modulus and bending strength testing stated by the ISO 5833.

The cylinders are placed between a flat noncompressible surface and the platen of the material testing machine (ZWICK/ROELL Z005) (see Figure 1(a)). The test machine is operated to produce a curve of displacement against load, using a constant crosshead speed of 20 mm/min stopping when the upper yield point has been passed or the cylinder obviously fractures. The compression strength for each cylinder is calculated using special software (TestExpert ZWICK/ROELL). The force applied until fracture or to reach the upper yield point is recorded and divided by the original cross-sectional area, in square millimeters, of the cylinder. The result is given in megapascals.

### 2.4. Bending Modulus and Bending Strength

The same procedure as mentioned for creating the PMMA cylinders is performed to create bars of 75 (±0.1) mm length, 10 (±0.1) mm width, and 3, 3 (±0.1) mm depth to determine the bending modulus and bending strength.

The bending modulus and bending strength of the bars are determined by means of a four-point bending test. The ISO 5833 exactly stipulates the apparatus in which the test is performed assuring comparable contact points of the platen to the cement bar (see Figure 1(b)). Operating the crosshead at a speed of 5 mm/min, the deflection under the specimen and the applied force are recorded until breakage. Bending modulus and bending strength are calculated using special software (TestExpert ZWICK/ROELL) taking the defined measurements, the deflection under certain loads, and the force at breaking point into account.

The above-mentioned tests are performed comparing PMMA without additional antibiotic, PMMA containing 1.225% per weight of gentamicin and PMMA with two different concentrations (1.225% and 7.5% per weight) of daptomycin.

## 3. Scanning Electron Microscopy

### 3.1. Preparation of Samples for Scanning Electron Microscopic Investigations

Pieces of bulky pure bone cement (PMMA) and of daptomycin-laden PMMA and commercial powders of PMMA and of daptomycin were prepared for morphological studies by scanning electron microscopy (SEM). Two different techniques were used to get access to the internal structure of the pieces of bulky bone cement: (i) individual pieces were frozen using liquid nitrogen to enhance their brittleness and subsequently mechanically fractured. The surface of fractures was used for the morphological studies with the SEM; (ii) individual pieces of bulky bone cement were cut at room temperature by using an ultramicrotome (Ultracut S from Reichert, Vienna, Austria). The cross-sections represent smooth block faces, which are well suited for the investigation with SEM.

Individual pieces of pure and laden PMMA, respectively, were glued to aluminum stubs with conductive carbon cement (Neubauer Chemikalien, Muenster, Germany) in optimum position for SEM imaging. Subsequently, the samples were sputter-coated with 15 nm gold to achieve a sufficient electric conductivity at the surface of the PMMA thereby avoiding electric charge-ups of the samples during imaging in the SEM.

The powders of PMMA and of daptomycin, respectively, were fixed to an aluminum support by means of melting glue (Tempfix, Plano GmbH, Wetzlar, Germany). Subsequently, the samples were sputter-coated with 15 nm gold to get sufficient electric conductivity at the surface.

### 3.2. Scanning Electron Microscopy

Scanning electron microscopic imaging at low and medium magnifications was performed with a field emission SEM S-800 (Hitachi Ltd., Tokyo, Japan) with 5 keV electrons in the secondary electron (SE) mode at a working distance of 5 mm. The SE micrographs were recorded digitally with 800 × 1000 pixels.

## 4. Elution Kinetics

The exothermic reaction of the PMMA there might cause a loss of the biological efficacy of the added antibiotic substance, for example, daptomycin.

**Figure 2:**
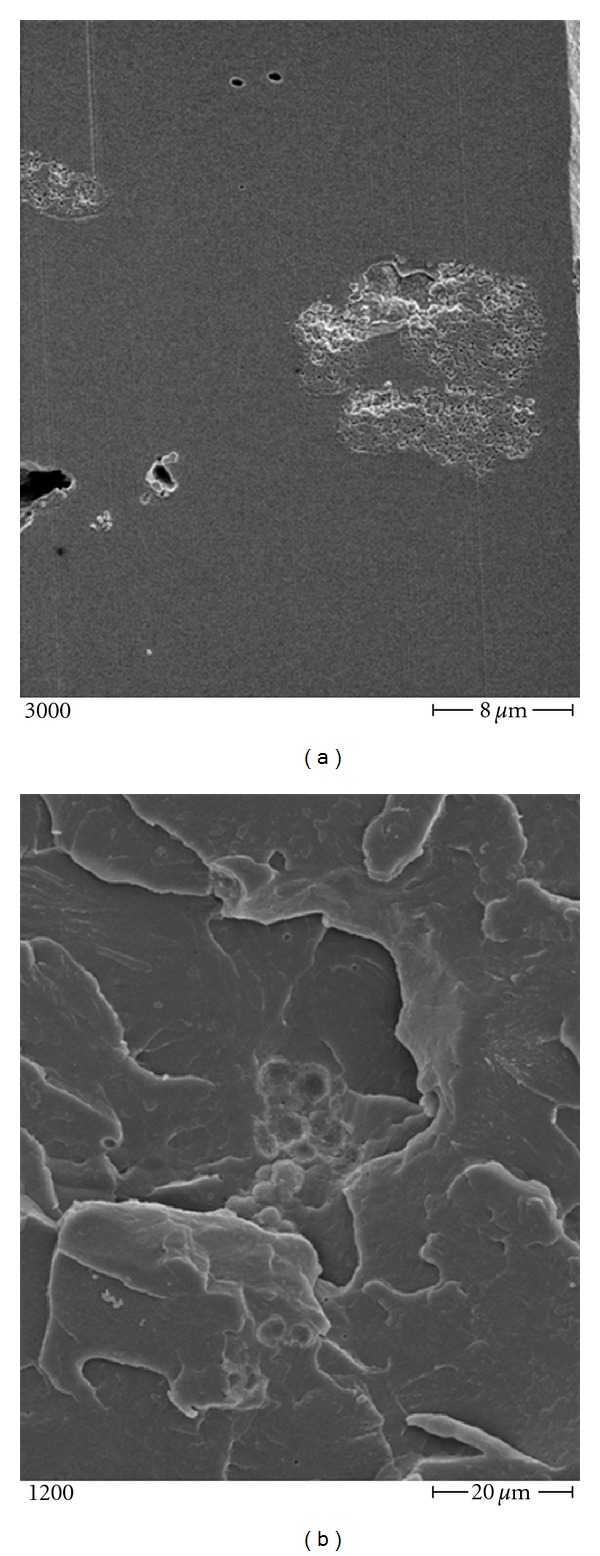
Scanning electron microscopy: daptomycin-laden PMMA at magnifications of (a) 3000 and (b) 1200.

Furthermore, the incorporation of the antibiotic substance into the homogenous structured PMMA (see Figures 2(a) and 2(b)) might trap the antibiotic, rendering it unable to elute to the surface and unable to show antibacterial activity. Giving rise to the need of checking the respective elution kinetics [20].

**Figure 3:**
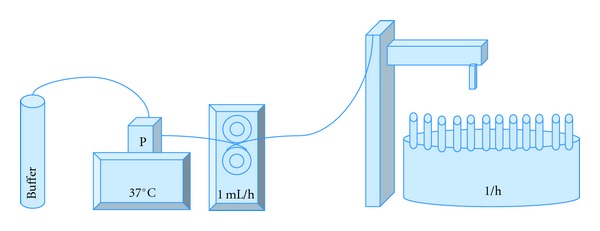
Continuous flow chamber modified according to Perry et al. [51].

Sterile PMMA cylinders of defined daptomycin concentrations (7.5% per weight) are produced as stated above and placed in a continuous flow chamber. This flow chamber according to Perry et al. [51] consists of (see Figure 3) a reservoir containing Ringer buffer, a container with the test-sample, and a fraction collector. A peristaltic pump flowing at a rate of 1 mL/h creates a constant flow along the antibiotic laden PMMA. The fraction collector gathered effluent every hour for 48 hours. Beforehand, a calibration curve was produced correlating defined antibiotic concentrations with resulting zones of inhibition on Mueller-Hinton agar plates. Taking this curve into account, the eluted concentration versus time is plotted on a graph showing the time-dependent elution of the antibiotic substance. Pathogens used were strains of *Enterococcus faecium* and *Staphylococcus epidermidis*.

## 5. Results

### 5.1. *In Vitro* Infection

Looking at the time-dependent decrease of intracellular CFUs when applying daptomycin to infected osteoblasts, a significant decrease was noted when comparing numbers for 20 h and 40 h application for the Cowan and the 49230 group. The same results were stated for the rest of the antibiotics used, showing time-dependent increase of intracellular efficacy. One exception was noted, showing no significant time-dependent CFU decrease for gentamicin in the TM 300 and the Cowan group (*P* = 0.35; *P* = 0.143).

**Figure 4:**
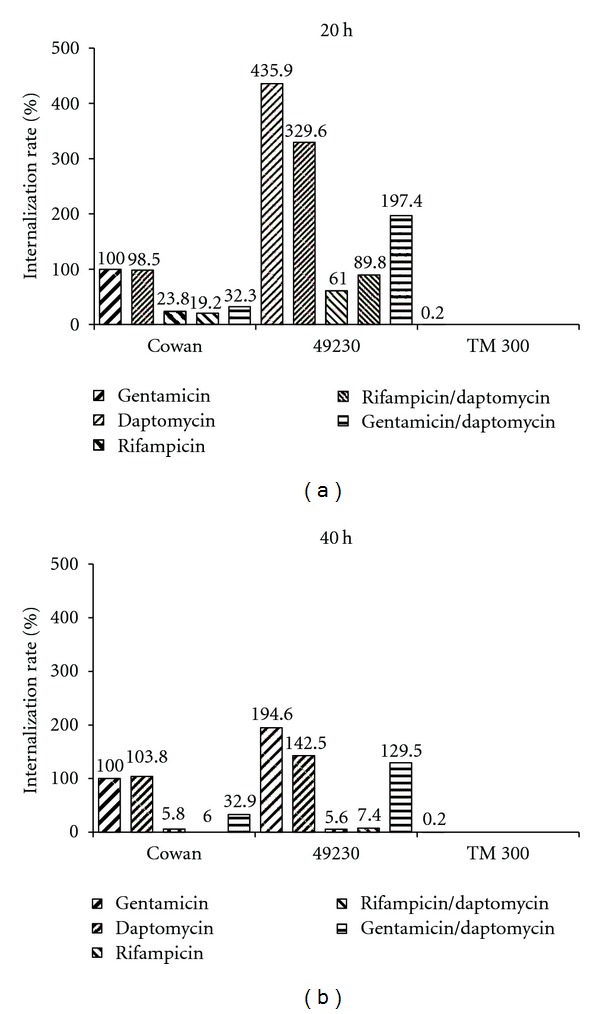
*In vitro* infection model—internalization rate for 20 h and 40 h of 3 different. *Staphylococcus* strains with application of different antibiotics. Invasiveness of Cowan with gentamicin set at 100%. TM 300 served as a negative control group.

For the duration of 20 h and for 40 h application, daptomycin showed neither within the two pathogen groups (Cowan; 49230) significant difference to gentamicin nor to the combination of daptomycin/gentamicin. The same was noted for gentamicin to the combination daptomycin/gentamicin. Significant decrease of intracellular CFUs was found in 20 h and 40 h use for rifampicin in single-use and in any combination (Ri + Dap, Ri + Genta) in comparison to daptomycin, gentamicin, or their combination. Addition of daptomycin or gentamicin to rifampicin showed no significant decrease in the number of intracellular CFUs for 20 h and for 40 h use compared to rifampicin in single use (see Figures 4(a) and 4(b)).

### 5.2. Biomechanics

There was no statistically significant change noted in compressive strength as well in bending modulus and bending strength of the PMMA when adding daptomycin of 1.225% per weight or 7.5% per weight compared to customary PMMA with or without antibiotic-agent.

### 5.3. Compressive Strength

**Figure 5:**
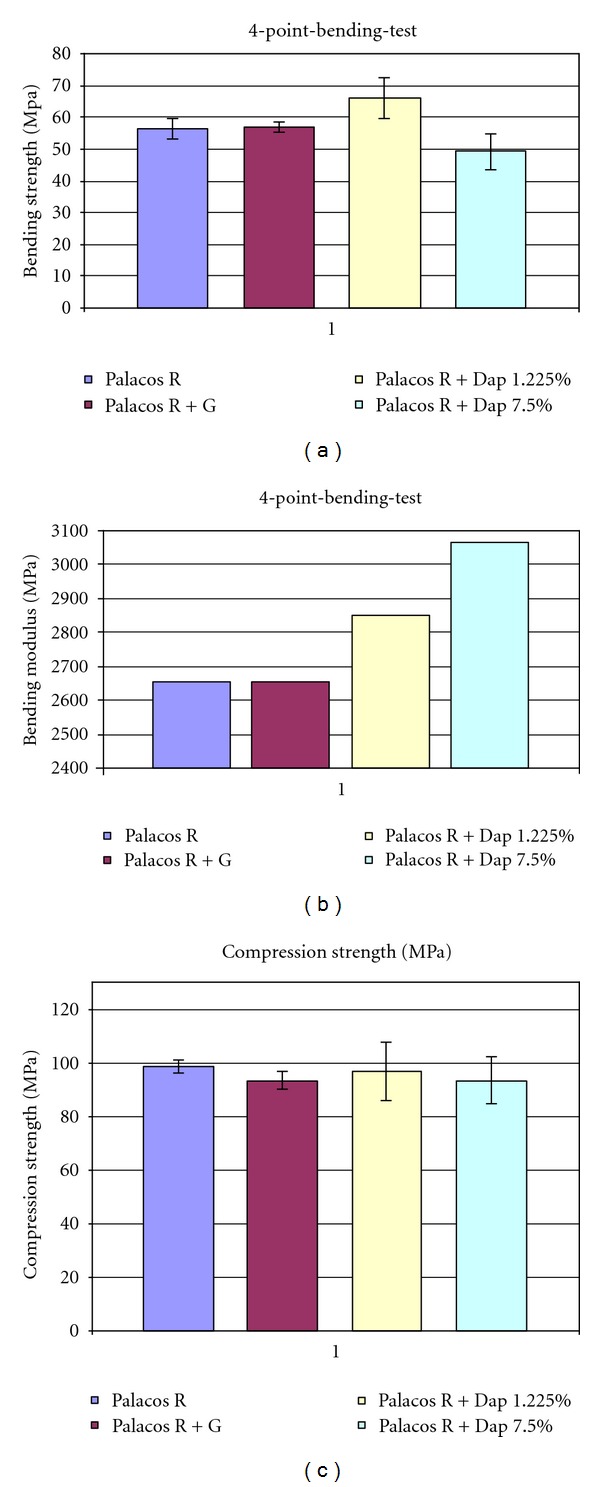
Average bending modulus, bending strength and compression strength.

Figure 5(a) shows the average compressive strength of the 4 different PMMA groups. The average maximum compressive strength in both daptomycin-laden groups was above 90 MPa well exceeding the by the ISO [50] required minimum strength of 70 MPa. Respectively, the same testing was performed for PMMA with gentamicin and PMMA without antibiotic supplement showing similar results exceeding stipulated requirements.

### 5.4. Bending Modulus and Bending Strength

Figures 5(b) and 5(c) show the values for bending strength and bending modulus of the 4 PMMA groups.

The bending modulus for PMMA with 1.225% and 7.5% per weight of daptomycin was with 2851.6 MPa and 3065.1 MPa well above the required value of 1800 MPa. The same applied for the values for PMMA with and without 1.225% per weight of gentamicin. In terms of bending strength, the lower concentrated daptomycin-PMMA groups showed sufficient strength at 66.2 MPa at required 50 MPa. With 7.5% per weight of daptomycin, the samples went with 49.3 MPa narrowly below the required limit.

### 5.5. Elution Kinetics

**Figure 6:**
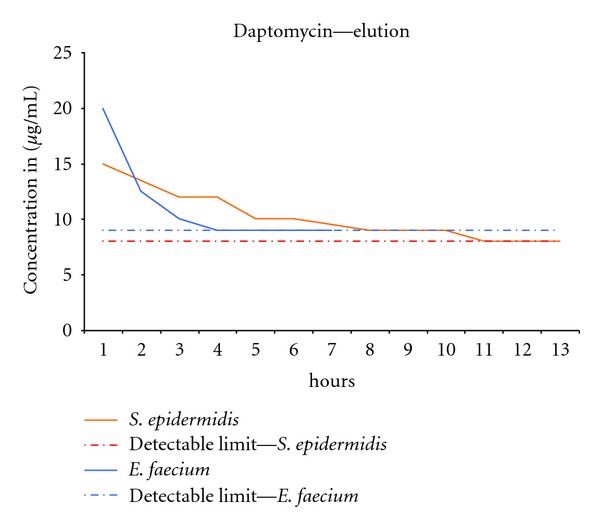
Elution kinetics for daptomycin measured by means of a bioassay with *S. epidermidis* and *E. faecium. *

Antimicrobial activity of the eluate of daptomycin-laden PMMA was detectable within the first hour of elution-testing. Its peak value of 15 *μ*g/mL in the *S. epidermidis* group and 20 *μ*g/mL in the *E. faecium* group was presented within the first hour. Further elution kinetics showed a sustained decrease until the detectable limit of 8 *μ*g/mL in the *S. epidermidis* group and 9 *μ*g/mL in the *E. faecium* group (see Figure 6) is reached. Elution kinetics was able to be examined for 13 hours in the *S. epidermidis* group and for 7 hours in the *E. faecium* group until reaching nondetectable values.

## 6. Discussion

With the constant rise of multiresistant pathogens causing complicated infections especially in trauma and orthopedic surgery, an urgent need for new agents exists. Daptomycin the first member of the new class of lipopeptide antibiotics adds a novel mechanism of antibacterial activity directed against gram-positive pathogens in this scenario.

When evaluating the possible applicability of new antibiotic agents, certain aspects especially in infection treatment and prophylaxis in trauma and orthopaedic surgery have to be taken into consideration.

With pathogens capable of persisting in osteoblasts, the intracellular activity of the antimicrobial agent is important. This study shows by comparing daptomycin with a commonly used antibiotic such as gentamicin, that there is no significant difference between these two agents concerning intracellular activity. Rifampicin or any combination containing rifampicin reduced the number of intracellular bacteria significantly more than gentamicin or daptomycin and their combination. Furthermore, no synergistic effects such as activity enhancement were seen by combining any of the used antibiotics rifampicin, gentamicin, or daptomycin compared to single-use. Because the *in vivo* single-use of rifampicin is not applicable due to rapid resistance induction, the addition of daptomycin might be an attractive alternative for treatment of complicated osteomyelitis. Furthermore, single application of rifampicin within PMMA is not possible due to preventing the PMMA from curing [45].

This testing did not account for *in vivo* circumstances such as protein binding, and so forth maybe causing activity change of the antibiotic substance. Further *in vivo* tests have to be performed to examine its characteristics more thoroughly.

Another important aspect is the ability of applying the agent locally, for example, in PMMA or other carrier to reach locally high concentrations of the active agent, whereas systemic effects are reduced. This ability is mainly determined by resulting structural and elution characteristics.

The ISO 5833 as the international standard for evaluating medical-used PMMA states the required biomechanical characteristics. Adding daptomycin within the range of physiological application to PMMA shows no significant negative effects on its biomechanical properties allowing sufficient strength for application, for example, as a spacer prostheses in treatment of implant-associated infection of the hip, as a spacer for treatment of complicated osteomyelitis (Figures 5(a), 5(b), and 5(c)). No statement can be given concerning behaviour under cyclic loading. Further tests will have to evaluate the daptomycin-laden PMMA under fatigue testing.

Elution characteristics of daptomycin from PMMA have been evaluated before by Hall et al. [52] and showed sufficient level at the sight of interest. This test-setup proved that after loading the PMMA with daptomycin it is still able to elute and to remain antibacterial active. To further determine elution characteristics in a more quantifiable way other than a Bioassay, instruments such as an HPLC present an effective alternative.

To determine elution characteristics of a certain agent out of a carrier by simulating intraosseous *in vivo* conditions within an *in vitro* test-setup, certain aspects such as intraosseous blood flow, movement, protein binding, and so forth, have to be taken into consideration. This test setup does not account for protein binding in serum, possible blood flow changes, and so forth, thus possibly resulting in a different antibiotic activity as *in vivo* conditions might allow. Furthermore, cyclic loading of the PMMA might have an influence of elution characteristics, which this test setup does not account for.

This study states that in terms of intracellular activity daptomycin shows no significant difference to commonly used antibiotics such as gentamicin in osteoblasts.

These *in vitro* results implicate that for complicated osteomyelitis and prosthetic joint infections due to multiresistant pathogens, susceptible to daptomycin, this novel antibiotic substance is an applicable alternative to currently used agents in orthopedic and trauma surgery.
